# Potential biomarkers for cerebral small vessel disease with cognitive impairment: a systematic review and meta-analysis

**DOI:** 10.3389/fnagi.2024.1475571

**Published:** 2025-01-07

**Authors:** Libin Liao, Weiquan Huang, Rongchao Ma, Xuan He, Moxi Su, Dujuan Sha

**Affiliations:** ^1^Nanjing Drum Tower Hospital Clinical College of Nanjing Medical University, Nanjing, China; ^2^Department of General Practice, Nanjing Drum Tower Hospital, Affiliated Hospital of Medical School, Nanjing University, Nanjing, China; ^3^State Key Laboratory of Pharmaceutical Biotechnology, Institute of Functional Biomolecules, Nanjing University, Nanjing, China

**Keywords:** cerebral small vessel disease, blood biomarker, cognitive impairment, dementia, meta-analysis

## Abstract

Cerebral small vessel disease (CSVD) is a common factor in age-related diseases such as stroke and dementia, and about half of dementia patients worldwide are caused by CSVD. CSVD-related cognitive impairment (CSVD-CI) affects more and more elderly people, resulting in economic losses and burdens on families and society. In recent years, circulating biomarkers have made breakthroughs and played an increasingly important role in the diagnosis, progression, and prognosis of CSVD-associated cognitive impairment, and are expected to be applied to the early clinical detection, diagnosis, and treatment of patients with cerebral small vessel disease. Through a systematic review and meta-analysis, this study aimed to assess the relationship between circulating factors and cognitive impairment associated with cerebral small vessel disease, especially the possibility of becoming the potential biomarkers for diagnosis. Articles published before November 2023 were searched in four databases, PubMed, Web of Science, Embase, and Cochrane Library, to identify all relevant studies reporting circulating markers in patients with CSVD. Twenty-nine articles out of 2,911 were finalized for this study. We meta-analyzed 2 or more articles that were jointly considered to be circulating biomarkers of CSVD-CI and summarized a total of 4 possible biomarkers: homocysteine (Hcy), high-sensitivity C-reactive protein (hs-CRP), lipoprotein-associated phospholipase A2 (Lp-PLA2), and neurofilament protein light chain (NfL). The results revealed that patients in the CSVD-related cognitive impairment group had significantly higher levels of Hcy and hs-CRP than those in the CSVD-without cognitive impairment group, whereas there was no statistically significant difference in Lp-PLA2 and NfL between the two groups. Therefore, Hcy, hs-CRP may be considered circulating markers of cognitive impairment associated with cerebral small vessel disease.

## 1 Introduction

Cerebral small blood vessels are important in regulating cerebral blood flow. The small vessel networks branch from the large cerebral vessels, and pial arterioles, initially perforating arterioles through the parenchyma, flowing into the capillary beds, and finally flowing into the venous (Cannistraro et al., 2019). Cerebral small vessel disease (CSVD) refers to intracranial vascular diseases with various pathologic processes that damage the cerebral small vessels, arterioles, capillaries, and small veins (Pantoni, 2010). The term “cerebral small vessel disease” describes multiple diseases affecting the small vessels of the brain, arterioles, capillaries, and small veins. The definition of the size of “small vessels” in CSVD can refer to the small arteries and small perforating arteries affected in CSVD, which have been described differently and varied greatly in published papers. The size of the lesion is described as follows: using the neuroimaging features of CSVD as the basis for classification, “recent small subcortical infarcts” lesions should have a maximum diameter of <20 mm, “Lacune of presumed vascular origin” of between 3 mm and 15 mm in diameter, “perivascular spaces” with a diameter is generally less than 3 diameters. “Cerebral microbleed” is usually 2–5 mm and sometimes up to 10 mm in diameter (Wardlaw et al., 2013). Currently, imaging classification is commonly used in the classification of CSVD, which is based on characteristic magnetic resonance features as follows: lacune, cerebral microbleed (CMB), white matter hyperintensities (WMH), enlarged perivascular spaces, and cerebral atrophy (Markus and de Leeuw, 2022). The pathological mechanisms of cerebral small vessel disease may be hypoperfusion/hypoxia, blood–brain barrier dysregulation, interstitial fluid/cerebrospinal fluid drainage disturbances, and vascular inflammation, which are the common pathological basis of vascular cognitive impairment and dementia and can lead to cognitive impairment and then dementia (Inoue et al., 2023). In addition to cognitive impairment, CSVD can also manifest as progressive dysphagia, dysarthria, depression, and emotional apathy (Inoue et al., 2023; Li et al., 2024). It is also strongly associated with renal function impairment, diabetic retinopathy, gait dysfunction, and falls (Eriksson et al., 2021; Ölmez et al., 2022; Sharma et al., 2023). Studies have reported that CSVD is the leading cause of vascular cognitive impairment (VCI), which accounts for at least 20–40% of all dementia patients (Hamilton et al., 2020; Rundek et al., 2022). Cognitive impairment affects the quality of patients’ survival and creates a heavy economic burden on society and families. Therefore, early diagnosis and intervention of CSVD-related cognitive impairment can reduce the incidence of VCI and the incidence of dementia. Currently, the diagnosis of CSVD and the degree of disease progression depends crucially on neuroimaging such as magnetic resonance imaging (MRI). At the same time, the evaluation of cognitive decline in CSVD is combined with physical indications such as relying on neuropsychological tests and total CSVD load (Wang J. et al., 2023). Therefore, the evaluation results are subjective and inaccurate, which is not yet sufficient for diagnosis and prognosis. Existing studies have shown that molecular biomarkers such as homocysteine (Hcy), High-sensitivity C-reaction protein (hs-CRP), Lipoprotein-Associated Phospholipase A2, and neurofilament light chain (NfL), may be strongly associated with cognitive impairment in CSVD (Cao and Sun, 2022; Liu et al., 2023; van Gennip et al., 2023). These accessible, simple, and convenient molecular biomarkers can improve disease diagnosis, and prognostic accuracy, and have important implications for the progression of the disease state and targeted therapy. Therefore, this article is a systematic review to discuss and analyze these potential molecular biomarkers for predicting cognitive impairment in CSVD.

## 2 Methods

### 2.1 Search strategy

We searched the following databases: Medline, PubMed, Web of Science, Embase, and Cochrane Library from inception to November 2023, the language was restricted to English. The protocol of this systematic review and Meta-analysis has been registered previously on PROSPERO, an international registration number is [CRD42024567632]. The databases screening and studies’ selection process followed the PRISMA (preferred reporting items for systematic reviews and meta-analyses).

We used different combinations of the following keyword search terms: (Cerebral Small Vessel Diseases OR Cerebral Microangiopathies OR Cerebral Microangiopathy OR Microangiopathies, Cerebral OR Microangiopathy, Cerebral) AND (Cognitive Dysfunctions OR Dysfunction, Cognitive OR Dysfunctions, Cognitive OR Cognitive Impairments OR Cognitive Impairment OR Impairment, Cognitive OR Impairments, Cognitive OR Cognitive Disorder OR Cognitive Disorders OR Disorder, Cognitive OR Disorders, Cognitive OR Mild Cognitive Impairment OR Cognitive Impairment, Mild OR Cognitive Impairments, Mild OR Impairment, Mild Cognitive OR Impairments, Mild Cognitive OR Mild Cognitive Impairments OR Cognitive Decline OR Cognitive Declines OR Decline, Cognitive OR Declines, Cognitive OR Mental Deterioration OR Deterioration, Mental OR Deteriorations, Mental OR Mental Deteriorations OR Cognitive defect OR cognitive defects OR cognitive disability OR cognitive dysfunction OR delirium OR dementia OR amnestic OR response interference) AND (Circulating OR Marker, Biological OR Biological Marker OR Biologic Marker OR Marker, Biologic OR Biological Markers OR Biologic Markers OR Markers, Biologic OR Biomarker OR Markers, Biological OR Markers, Immunologic OR Immune Markers OR Markers, Immune OR Marker, Immunologic OR Immunologic Markers OR Immune Marker OR Marker, Immune OR Immunologic Marker OR Serum Markers OR Markers, Serum OR Marker, Serum OR Serum Marker OR Surrogate Endpoints OR Endpoints, Surrogate OR Surrogate End Point OR End Point, Surrogate OR Surrogate End Points OR End Points, Surrogate OR Surrogate Endpoint OR Endpoint, Surrogate OR Markers, Clinical OR Clinical Markers OR Clinical Marker OR Marker, Clinical OR Viral Markers OR Markers, Viral OR Viral Marker OR Marker, Viral OR Biochemical Marker OR Markers, Biochemical OR Marker, Biochemical OR Biochemical Markers OR Markers, Laboratory OR Laboratory Markers OR Laboratory Marker OR Marker, Laboratory OR Surrogate Markers OR Markers, Surrogate OR Marker, Surrogate OR Surrogate Marker OR Plasmas OR Blood Plasma OR Blood Plasmas OR Plasma, Blood OR Plasmas, Blood OR Fresh Frozen Plasma OR Fresh Frozen Plasmas OR Frozen Plasma, Fresh OR Frozen Plasmas, Fresh OR Plasma, Fresh Frozen OR Plasmas, Fresh Frozen OR Serums OR Blood Serum OR Serum, Blood OR marker OR markers).

### 2.2 Inclusion and exclusion criteria

The inclusion criteria of the meta-analysis were as follows:(1) cohort, case–control or cross-sectional studies investigating blood biomarkers in patients with cerebral small vessel disease and assessing their value in the progression, diagnosis, and prognosis of cognitive impairment in cerebral small vessel disease; (2) CT/MRI-based diagnosis of cerebral small vessel disease events in patients ≥18 years of age; (3) measures of physical and cognitive outcomes using validated motor and cognitive assessment scales (e.g., the NIHSS, mRS and/or mBI, MMSE, MoCA); (4) studies which reported measures for the prognostic value of the described biomarkers, e.g., sensitivity, specificity, area under the curve (for dichotomized outcomes), or correlation coefficients between the biomarkers and results of the scales assessed physical and cognitive outcomes.

We excluded from the searched studies: (1) irrelevant to the topic exploring the relationship between the blood biomarkers and cognitive function in CSVD. (2) Cellular or animal experiments, reviews, conference proceedings, commentaries, Meta-analyses, case reports, etc. (3) Non-cerebral small vessel disease patients. (4) Comparison of non-CSVD cognitive impairment and normal CSVD cognitive function, or comparison of blood markers under non-different CSVD loads. (5) Studies without available data can be extracted, and studies without a control group.

As long as the above conditions were met, there are no restrictions on gender, ethnicity, or age range were placed on the population of the study.

### 2.3 Data extraction

Two reviewers work independently to extract the following data from eligible studies: the first author, the year of publication, country, study design, type and sample size of study and control groups, the type of specimen, and measurement tool used for evaluating cognitive function; and biomarker information, including the type of specimen and type and level of potential biomarkers. Any difference in the included studies was solved by discussion with another author. The screening and retrieving process for all articles was conducted in Endnote 20.2.1.

### 2.4 Quality assessment

Two authoritative quality assessment tools were used to assess all the included studies, The Newcastle Ottawa quality assessment scale (NOS) criteria was used to evaluate the cohort studies or case–control studies’ quality, while the cross-sectional study used The Agency for Healthcare Research and Quality (AHRQ) evaluation standards. In the NOS scale, both cohort and case–control studies were evaluated on 3 dimensions which are selection, comparability, and assessment. The 3 dimensions were subdivided into 9 points assessing the two types of the included papers, with minor differences, but broadly consistent. The highest score on the NOS scale is nine points, an NOS score ≥ 7 is considered a high-quality study, less than 3 points is defined as a low-quality study, and those scores between 4 and 6 are the median-quality articles (Yin et al., 2024). The AHRQ methodology checklist was used to evaluate the quality of the cross-sectional studies across 11 domains. Score ‘0’ is attributed to domains considered as ‘No’, score ‘1’ to domains considered as ‘Yes’, whereas ‘not sure’ represent that we could not find sufficient evidence to assess (Zhang S. et al., 2023). High-quality articles receive six to seven points on AHRQ’s cross-sectional study evaluation standard, the median quality scores achieve four to five points, and the low-quality articles receive less than four points (Gao et al., 2023).

### 2.5 Statistical analysis

For the meta-analysis, the standardized mean difference (SMD) in the potential biomarkers for evaluating CSVD-CI was analyzed between patients with and without CSVD-CI analysis using Review Manager 5.4 software. Heterogeneity was tested by Q-test (*α* = 0.1) and evaluated with I^2^ statistic. (When *p* > 0.1 and I^2^ ≤ 50%, the data were combined using a fixed-effects model, and vice versa, a random-effects model was used) (Mathew, 2022). If there was no statistical heterogeneity among the results of the studies, the fixed-effects model was used for Meta-analysis; if there was statistical heterogeneity among the results of the studies, the random-effects model was used for Meta-analysis after excluding the influence of obvious clinical and methodological heterogeneity. If possible, a sensitivity analysis will be used to examine further the impact of individual studies on the overall outcomes and analyze whether the results are stable and reliable (Lee, 2018).

## 3 Results

### 3.1 Study selection

**Figure 1:**
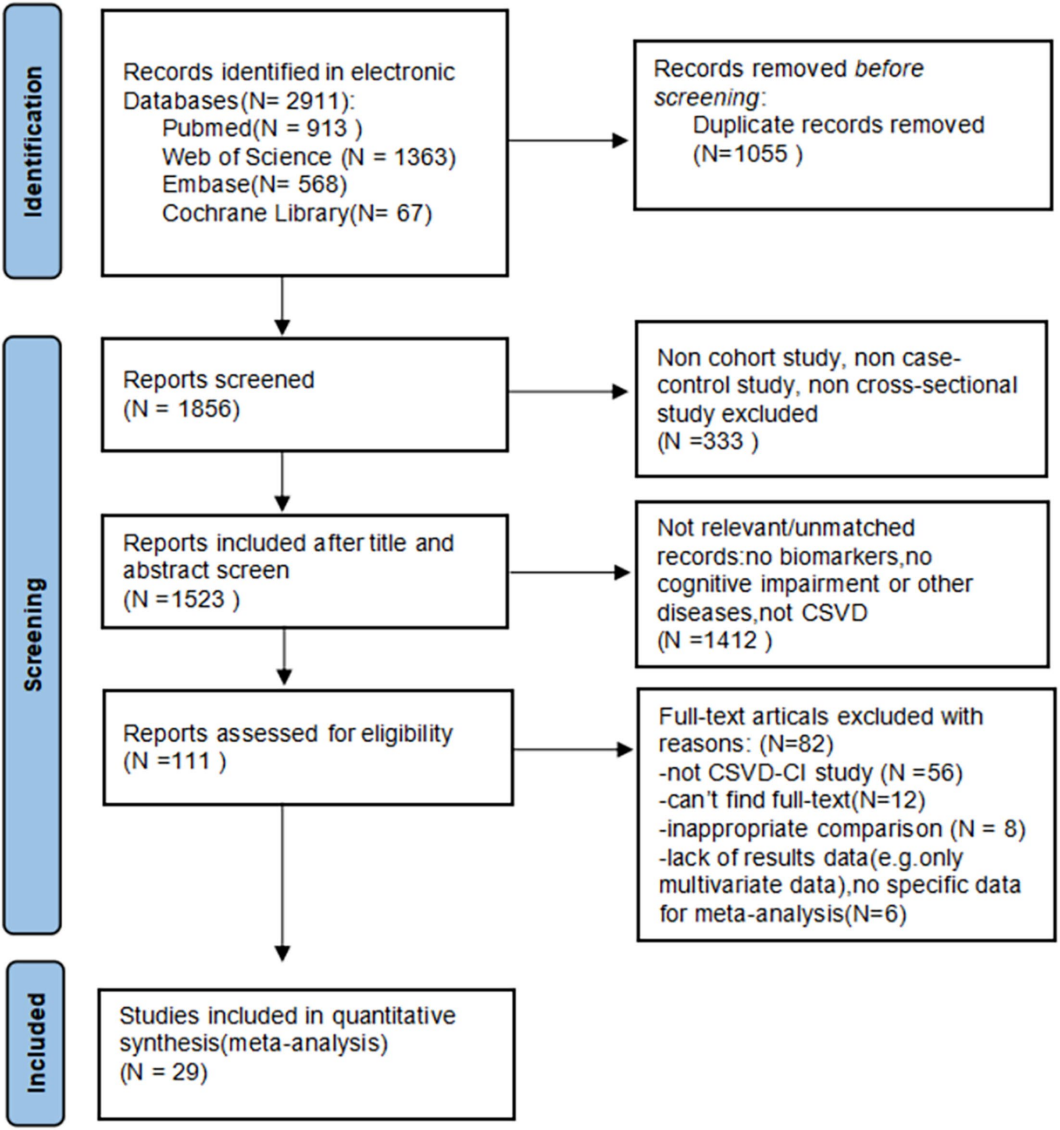
Flow diagram of the study selection.

Through the database searches, according to the established search strategy, a total of articles 2,911 were selected for subsequent filtering. Of these, 1,055 duplicate articles were excluded. After checking the titles and abstracts of each paper, 333 articles were excluded because of cellular and animal experiments, reviews, conference articles, Meta-analysis, case reports, and other inconsistent literature types. Also, 1,412 articles that were irrelevant to the topic were excluded. The remaining 111 papers were browsed in full text, and 82 articles were excluded for the following reasons: 8 articles were excluded because of inappropriate control groups and failure to reflect the relationship between circulating markers and CSVD; 56 articles were excluded because the disease is not CSVD; 12 articles were excluded because the full text was not found, and 6 articles lack of results or no specific data for meta-analysis. Twenty-nine studies met our inclusion and exclusion criteria and were included in our review. A flow sheet of the literature selection process can be visualized in Figure 1.

### 3.2 Characteristics of the included studies reporting potential biomarkers for CSVD-CI

The characteristics of the included studies are summarized in Table 1. The included studies in which 28 (96.55%) were articles from the last 10 years and 23 articles (79.31%) were published in the last 5 years. In our study, 22 articles were from China, which makes up a large portion of the study sources. There were also researchers and scholars from India (Prabhakar et al., 2017), the UK (Harshfield et al., 2022), the Netherlands (Hilal et al., 2017; van Gennip et al., 2023), Japan (Wada et al., 2011), the USA (Yoo et al., 2020), and Singapore (Salai et al., 2023) who explored biomarkers of CSVD-CI. Six thousand four hundred and sixty patients were included in the control group in which mainly consisted of healthy people, patients with normal cognitive function, and patients with normal cognitive function in CSVD. Five thousand eight hundred and forty-one patients were included in the experimental group, mostly patients with CSVD-CI and patients with mild cognitive impairment and moderate-to-severe cognitive impairment were also included in experimental group (Liu et al., 2023; Tao et al., 2022; Zhang W. et al., 2023; Zhao et al., 2021; Zhu et al., 2019). Some of the patients categorized based on characteristic magnetic resonance features of CSVD, such as cerebral microbleeds (CMBs), and white matter hyperintensity (WMHs) were included in the experimental group as well (Hilal et al., 2017; Wada et al., 2011). Experimental groups were also grouped based on the concentration of biomarkers explored (Qiu et al., 2022; Yang et al., 2019). The cohort studies included in this study mostly used baseline as the control group and patients who showed changes in cognitive function after follow-up as the experimental group (Harshfield et al., 2022; Hilal et al., 2017; Huang et al., 2023; Salai et al., 2023; van Gennip et al., 2023). In addition, we also counted the specimen sources, the biomarker specimens were mainly from 3 categories: serum, plasma, and blood. Serum was the most frequent biomarker specimen, amounting to 20 specimens. The rest of the specimens were plasma or blood, except for one study in which platelets were used as the specimen (Xiao et al., 2023). MMSE and MOCA are the common outcome measurement tools used for evaluating cognitive impairment in our study. In addition, we also found three articles using Clinical Dementia Rating (CDR) (Bian et al., 2023; Yoo et al., 2020; Zhao et al., 2021), three articles using Trail Making Test (TMT) (Chen et al., 2023; Qu et al., 2023; Zhang W. et al., 2023) as scale to assess cognitive function. Two articles did not mention which outcome measure tools was used (Prabhakar et al., 2017; Salai et al., 2023). The prospective study had a follow-up period, whereas the retrospective study had no follow-up. Potential biomarkers are listed in Table 1 (Tao et al., 2022; Bian et al., 2023; Cao and Sun, 2022; Cao et al., 2019; Chen et al., 2023; Cui et al., 2019; Harshfield et al., 2022; Hilal et al., 2017; Huang et al., 2023; Kang et al., 2021; Liu et al., 2023; Liu and Fan, 2023; Prabhakar et al., 2017; Qiu et al., 2022; Qu et al., 2023; Salai et al., 2023).

**Table 1:** Summary of the 29 selected studies reporting potential biomarkers for CSVD with CI.

**Study and author**	**Study groups**		**Control**		**Specimen**	**Potential biomarkers**	**Reference**
	**Subjects**	**Number**	**Subjects**	**Number**			
Laifang Bian et al. (2023)	CSVD-CI	140	CSVD without CI	79	Serum	PKM2	Bian et al. (2023)
Li Cao et al. (2022)	CSVD-CI	90	Healthy group	92	Serum	Hcy/hsCRP/Hsp70	Cao and Sun (2022)
			CSVD without CI	87			
Yuqin Cao et al. (2019)	CSVD-CI	26	Healthy group	51	Serum	3-NT	Cao et al. (2019)
			CSVD without CI	29			
Xiaohan Chen et al. (2022)	CSVD-CI	52	CSVD without CI	48	Serum	NFL	Chen et al. (2023)
Ying Cui et al. (2019)	CSVD-CI	194	Healthy group	70	Serum	Lpa, FIB, D-D	Cui et al. (2019)
			CSVD without CI	106			
Harshfield Eric L et al. (2022)	symptomatic SVD with CI (change)	624	symptomatic SVD (baseline)	624	Serum	Creatine, Unsaturated fatty acids: FA, Glycerophospholipids (5:Diacylglycerophosphocholines: PC (16:0/20:5), PC (16:0/18:1)_1, PC (14:0/18:2), Diacylglycerophosphocholines: PC (16:0/20:5), PC (16:0/18:1)_1, PC (14:0/18:2)), Sphingolipids (17: Ceramide phosphocholines (sphingomyelins): SM (d18:2/24:0), SM (d18:2/23:0), SM (d18:2/22:0), SM (d18:1/24:0), SM (d18:1/23:0), SM (d18:1/22:0), SM (d16:1/24:0), SM (d16:1/22:0), SM (d18:2/24:1), Hexosylceramides: HexCer (d16:1/24:0), N-acylsphingosines (ceramides): Cer (d41:1)/Cer (d18:1/23:0)/Cer (d17:1/24:0), Cer (d19:1/24:0), Cer (d18:2/24:0), Cer (d18:1/25:0), Cer (d18:1/24:0), Cer (d16:1/24:0), Cer (d16:1/22:0), Cholesterol, Paraxanthine, Caffeine, HDL-4_Apo-A2)	Harshfield et al. (2022)
Hilal Saima et al. (2017)	The cohort with Cerebral microbleeds	353	baseline	1,201	plasma	Aβ1-38, Aβ1-40 and Aβ1-40/Aβ1-42 ratio	Hilal et al. (2017)
			the Cohort without Cerebral microbleeds	389			
Ronghui Huang et al. (2023)	CSVD-CI (change)	61	CSVD (baseline)	188	serum	NLRP3(nucleotide-binding oligomerization domain (NOD)-like receptor protein 3)	Huang et al. (2023)
Jingwen Kang et al. (2021)	CSVD-CI	117	CSVD without CI	99	serum	IGF-1	Kang et al. (2021)
Lu Liu et al. (2022)	mild cognitive impairment	72	normal cognition	105	serum	Lp-PLA2	Liu et al. (2023)
	moderate–severe cognitive impairment	36					
Shasha Liu et al. (2022)	CSVD	85	healthy control	30	serum	FT3, Apelin	Liu and Fan (2023)
Puttachandra Prabhakar et al. (2017)	small vessel VaD	204	healthy control	200	plasma	miR-409-3p, miR-376a-3p, miR-32-5p, miR-502-3p, miR-486-5p, miR-451a, miR-363-3p	Prabhakar et al. (2017)
Qianwen Qiu et al. (2022)	higher cortisol group ≥13.6	79	Lower cortisol group <13.6	79	serum	cortisol	Qiu et al. (2022)
Wensheng Qu et al. (2022)	CSVD	134	case control	702	Plasma	tAβ42, oAβ42	Qu et al. (2023)
Salai Kaung H. T. et al. (2023)	CI no dementia	103	No CI	93	Serum	TNF-R1	Salai et al. (2023)
Tao Xi et al. (2021)	mild VCI	103	normal cognition	98	blood	Erythrocytes, Hb, prealbumin, hs-CRP, RBP	Tao et al. (2022)
	severe VCI	101					
Gennip April C. E. van et al. (2023)	the Cohort	1,069	baseline	1,200	Plasma	NfL, GFAP, t-tau	van Gennip et al. (2023)
Wada Manabu et al. (2010)	Grades of White Matter Lesion (Grade 1)	300	Grades of White Matter Lesion (Grade 0)	203	Plasma	Fibrinogen	Wada et al. (2011)
	Grades of White Matter Lesion (Grade 2)	106					
	Grades of White Matter Lesion (Grade 3)	58					
Fei Wang et al. (2017)	CSVD-CI	80	CSVD without CI	92	Serum	S100β	Wang F. et al. (2017)
			healthy control	105			
Jin Wang et al. (2022)	CSVD-CI	47	CSVD without CI	59	Serum	VEGF	Wang J. et al. (2022)
Minghua Wang et al. (2023)	CSVD-CI	72	CSVD without CI	67	Serum	HMGB1	Wang M. et al. (2023)
Yanhong Wu et al. (2018)	CSVD	102	No CSVD	56	Serum	UA	Wu et al. (2018)
Yining Xiao et al. (2022)	CSVD-CI	133	CSVD without CI	68	platelet count × neutrophil count/lymphocyte count	SII	Xiao et al. (2023)
Yanfang Yang et al. (2019)	CSVD (15 μmol/L ≤ Hcy <20 μmol/L)	44	CSVD (Hcy <15 μmol/L)	32	Plasma	Hcy	Yang et al. (2019)
	CSVD (Hcy ≥20 μmol/L)	21					
Yoo Jun Sang.et al. (2020)	with Microbleeds	161	without Microbleeds	658	Plasma	Hcy	Yoo et al. (2020)
Wei Zhang et al. (2023)	CSVD with mild CI	54	healthy control	40	Serum	YKL-40	Zhang W. et al. (2023)
			CSVD without CI	56			
Jianhua Zhao et al. (2017)	CSVD-CI	40	CSVD without CI	40	Serum	MMP-9, Hcy	Zhao et al. (2022)
Weina Zhao et al. (2021)	CSVD-CI	40	normal controls (NC)	35	Blood	Exosomal miRNA-223-3p	Zhao et al. (2021)
			CSVD	38			
Shuzhen Zhu et al. (2019)	CSVD with mild CI	30	normal cognition	46	Serum	Lp-PLA2, SOD	Zhu et al. (2019)
	CSVD with severe CI	11					

3-NT: 3-nitrotyrosine, Aβ40: Amyloid-beta protein 40, Aβ42: Amyloid-beta protein 42, CI: cognitive impairment, D-D: D-dimer, FT3: free triiodothyronine, GFAP: glial fibrillary acidic protein, HC: Health control, Hcy: homocysteine, HMGPB1: High Mobility Group Protein B1, hsCRP: high-sensitivity C-reactive protein, Hsp70: heat shock protein 70, IGF-1: insulin-like growth factor-1, Lpa: Lipoprotein a, Lp-PLA2: lipoprotein-associated phospholipase A2, MMP-9: atrix metalloproteinase-9, NFL: Neurofilament light chain, PKM2: pyruvate kinase muscle isozyme 2, RBP: retinol binding protein, SII: systemic immune-inflammation index, SOD: superoxide dismutase, TNF-R1: Tumor necrosis factor-receptor 1, t-tau: total-tau, VEGF: vascular endothelial growth factor, UA: Uric Acid, YKL-40: chitinase-3 like protein-1 (CHI3L1) or human cartilage glycoprotein 39.

### 3.3 Classification of possible circulating markers of cognitive dysfunction

The potential biomarkers identified were categorized into 7 categories: blood and vascular functions, inflammatory and immune functions, metabolic functions, neuronal function, kidney function, hormones, and others.

Among potential biomarkers, homocysteine, D-dimer, retinol binding protein, fibrinogen, vascular endothelial growth factor, pyruvate kinase muscle isoenzyme 2, 3-nitrotyrosine, high-sensitivity C-reactive protein, heat shock protein 70, lipoprotein a, nucleotide-binding oligomerization structural domain (NOD)-like receptor protein 3 (NLRP3), lipoprotein-associated phospholipase A2, tumor necrosis factor-receptor 1, High Mobility Group Protein B1, systemic immune-inflammation index, chitinase-3 like protein-1(CHI3L1) or human cartilage glycoprotein 39 (YKL-40), matrix metalloproteinase-9, partially unsaturated fatty acids, Glycerophospholipids, Sphingolipids, high-density lipoprotein 4, neurofilament light chain, amyloid beta protein 1–38, Amyloid beta protein 1–40, Amyloid beta protein 1-40/Amyloid beta protein 1–42 ratio, total-tau, S100β, creatinine, uric acid, microRNA −32-5p, microRNA −486-5p, microRNA -451a, microRNA −363-3p, microRNA −223-3p levels increased among patients with CSVD-CI.

In addition, levels of biomarkers were decreased in erythrocytes, hemoglobin, prealbumin, lipoprotein-associated phospholipase A2, superoxide dismutase, insulin-like growth factor-1, paraxanthine, caffeine, free triodothyronine, cortisol, and microRNA-409-3p, microRNA-376a-3p. See the summary table for details (Table 2).

**Table 2:** Changes in the potential blood biomarkers for CSVD-CI.

**Category**	**Level**	**Potential biomarkers**
Blood and vascular functions	Increase	Hcy (Cao and Sun, 2022, Yang et al., 2019, Yoo et al., 2020, Zhao et al., 2022), D-D (Cui et al., 2019), RBP (Tao et al., 2022), fibrinogen (Cui et al., 2019, Wada et al., 2011), VEGF (Wang J. et al., 2022)
	Decrease	Erythrocytes (Tao et al., 2022), Hb (Tao et al., 2022), prealbumin (Tao et al., 2022)
Inflammatory and immune functions	Increase	PKM2 (Bian et al., 2023), 3-NT (Cao et al., 2019), hsCRP (Cao and Sun, 2022, Tao et al., 2022), Hsp70 (Cao and Sun, 2022, Tao et al., 2022), Lpa (Cui et al., 2019), NLRP3 (Huang et al., 2023), Lp-PLA2 (Liu et al., 2023), TNF-R1 (Salai et al., 2023), HMGB1 (Wang M. et al., 2023), SII (Xiao et al., 2023), YKL-40 (Zhang W. et al., 2023), MMP-9 (Zhao et al., 2022)
	Decrease	Lp-PLA2 (Zhu et al., 2019), SOD (Zhu et al., 2019)
Metabolic function	Increase	Unsaturated fatty acids (Harshfield et al., 2022), Glycerophospholipids (Harshfield et al., 2022), Sphingolipids (Harshfield et al., 2022), HDL-4_Apo-A2 (Harshfield et al., 2022)
	Decrease	IGF-1 (Kang et al., 2021), Paraxanthine (Harshfield et al., 2022), Caffeine (Harshfield et al., 2022)
Neuronal function	Increase	NfL (Chen et al., 2023, van Gennip et al., 2023), Aβ1-38 (Hilal et al., 2017), Aβ1-40 (Hilal et al., 2017), Aβ1-40/Aβ1-42 ratio (Hilal et al., 2017), t-tau (van Gennip et al., 2023), tAβ42 (Qu et al., 2023), oAβ42 (Qu et al., 2023), S100β (Wang F. et al., 2017)
Kidney function	Increase	Creatine (Harshfield et al., 2022), Uric Acid (Wu et al., 2018)
Hormone	Decrease	FT3 (Liu and Fan, 2023), cortisol (Qiu et al., 2022)
Others	Increase	miR-32-5p (Prabhakar et al., 2017), miR-502-3p (Prabhakar et al., 2017), miR-486-5p (Prabhakar et al., 2017), miR-451a (Prabhakar et al., 2017), miR-363-3p (Prabhakar et al., 2017), miRNA-223-3p (Zhao et al., 2021)
	Decrease	miR-409-3p (Prabhakar et al., 2017), miR-376a-3p (Prabhakar et al., 2017)

3-NT: 3-nitrotyrosine, Aβ40: Amyloid-beta protein 40, Aβ42: Amyloid-beta protein 42, D-D: D-dimer, FT3: free triiodothyronine, GFAP: glial fibrillary acidic protein, HC: Health control, Hcy: homocysteine, HMGPB1: High Mobility Group Protein B1, hsCRP: high-sensitivity C-reactive protein, Hsp70: heat shock protein 70, IGF-1: insulin-like growth factor-1, Lpa: Lipoprotein a, Lp-PLA2: lipoprotein-associated phospholipase A2, MMP-9: atrix metalloproteinase-9, NFL: Neurofilament light chain, PKM2: pyruvate kinase muscle isozyme 2, RBP: retinol binding protein, SII: systemic immune-inflammation index, SOD: superoxide dismutase, TNF-R1: Tumor necrosis factor-receptor 1, t-tau: total-tau, VEGF: vascular endothelial growth factor, UA: Uric Acid, YKL-40: chitinase-3 like protein-1 (CHI3L1) or human cartilage glycoprotein 39.

### 3.4 Literature quality assessment

The 29 included studies revealed that seven studies are cross-sectional study assessed with the AHRQ evaluation standard, and all seven included studies were considered of high quality. There are 17 case–control studies and six cohort studies, all of the remaining 21 studies’ quality assessment resulted in NOS of 6 for five studies, 7 for 12 studies, 8 for five studies, and 9 for one study. To sum up, there were 5 moderate-quality studies and 24 high-quality studies. Specific scores for each study are shown in Supplementary Tables S1A–C.

### 3.5 Meta-analysis results of the potential biomarkers of CSVD-CI and publication bias

**Figure 2:**
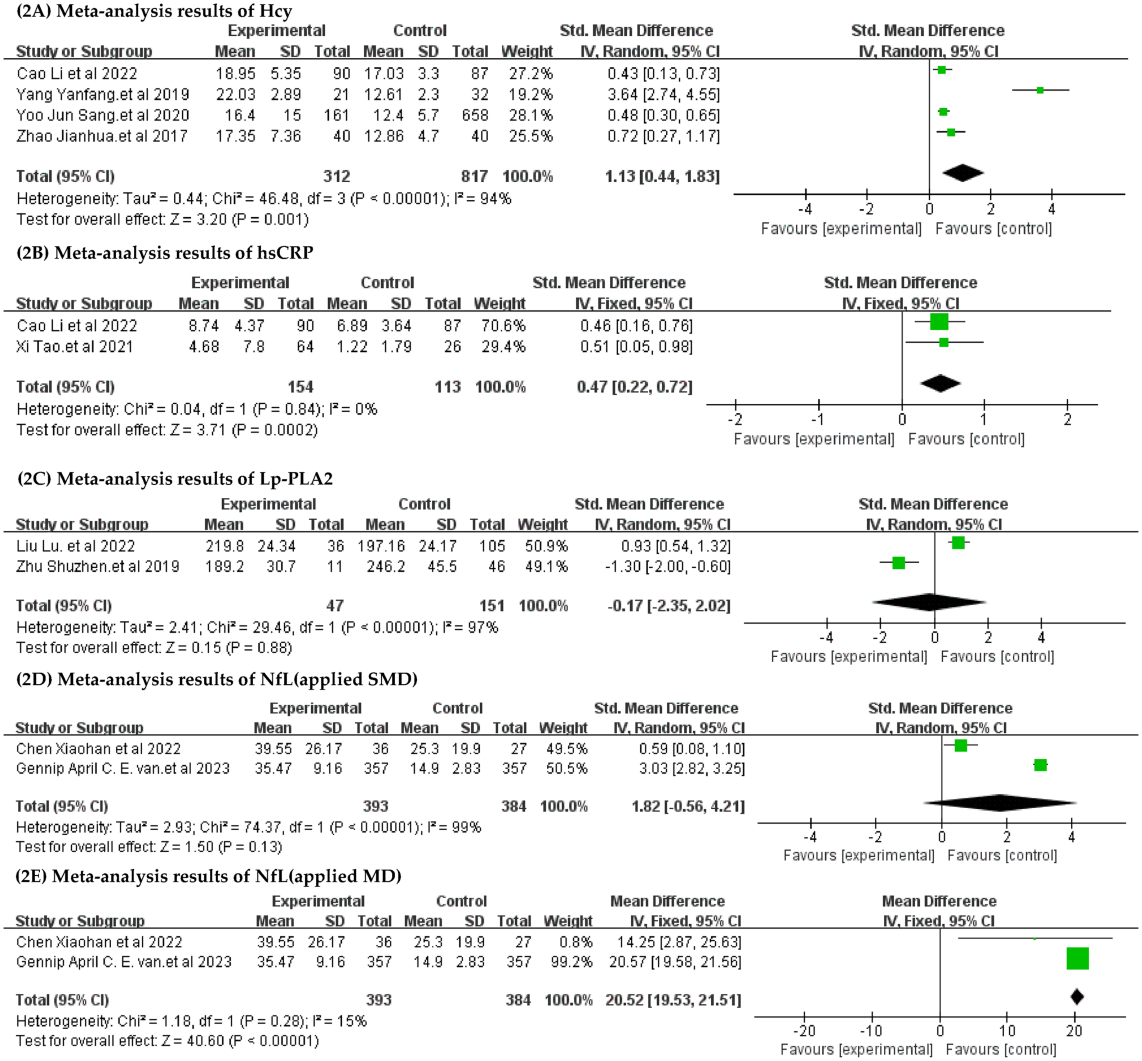
Forest plots for potential biomarkers. **(A)** Meta-analysis results of Hcy. **(B)** Meta-analysis results of hsCRP. **(C)** Meta-analysis results of Lp-PLA2. **(D)** Meta-analysis results of NfL,applied standardized mean difference (SMD) to the statistical analysis. **(E)** Meta-analysis results of NfL,applied mean difference (MD) for statistical analysis. Hcy: Homocysteine; hsCRP:high sensitivity C-Reactive protein;Lp-PLA2:Lipoprotein-associated phospholipase A2; NfL: Neurofilament light chain.

Below are the results of the meta-analysis of 2 or more papers that commonly support a potential biomarker for CSVD-CI (Figure 2):

1. Meta-analysis results of Hcy: As shown in Figures 2A, 4 articles were included, and the heterogeneity was significant (I2 = 94%, *p* < 0.00001); thus, we used the random-effects model. The Hcy level significantly differed between the CSVD-CI and the CSVD without CI groups [SMD = 1.13, 95% confidence interval (CI): 0.44–1.83, *p* = 0.001].
2. Meta-analysis results of hsCRP: 2 articles were included. The fixed-effects model was applied (I^2^ = 0%, *p* = 0.84), The hsCRP level significantly differed between the CSVD-CI and the CSVD without CI groups [SMD = 0.47, 95% confidence interval (CI): 0.22–0.72, *p* = 0.0002] (Figure 2B).
3. Meta-analysis results of Lp-PLA2: 2 articles were included, and the heterogeneity was significant (I^2^ = 97%, *p* < 0.00001); thus, we used the random-effects model. The Lp-PLA2 level did not differ between the CSVD-CI and the CSVD without CI groups [SMD = −0.17, 95% confidence interval (CI): −2.35 to 2.02, *p* = 0.88] (Figure 2C).
4. It is worth noting that the Meta-analysis results of NfL: 2 articles were included, when we applied standardized mean difference (SMD) to the statistical analysis, the heterogeneity was significant (I^2^ = 99%, *p* < 0. 00001), thus, we used the random-effects model. The NfL level did not differ between the CSVD-CI and the CSVD without CI groups (SMD = 1.82, 95% CI: −0.56 to 4.21, *p* = 0.13) (Figure 2D). But when we applied mean difference (MD) for statistical analysis, it was a low heterogeneity using a fixed effect model (I^2^ = 15%, p = 0. 28), The results showed a statistically significant difference between patients in the CSVD-CI and the CSVD without CI groups (MD = 20.52, 95% CI: 19.53–21.51, *p* < 0.00001) (Figure 2E).

Although SMD was uniformly used for statistical analysis in this study, we also applied MD for statistical analysis in three other biomarkers. However, we only found NfL to be significantly different under the two statistical units, so it is hereby noted. Therefore, the statistical significance of NfL in patients with CSVD-CI needs to be further explored.

We also found that there were also 2 articles supporting the biomarkers for CSVD-CI: D-dimer (Kong et al., 2022) and uric acid (Wang T. et al., 2017), because the articles were without specific data and were only correlation studies, the relevant data could not be extracted, therefore the articles were not included, as meta-analysis could not be performed. Thus, this article analyzed the above four markers. To test the publication bias of the included study, we used a visual funnel plot in Hcy analysis because it had four studies, the largest number of studies among our four biomarkers. The analysis showed the presence of publication bias in Hcy analysis as inferred by the symmetry of the funnel plots, individual studies tilted to the left side of the funnel (Supplementary Figure S2). The sensitivity analysis revealed that the results obtained from random-effect analyses from the articles were stable and reliable. In other words, the effect size after excluding individual studies was in good agreement with the total combined effect size and confidence interval [SMD = 1.13, 95% confidence interval (CI): 0.44–1.83, *p* = 0.001], indicating that our results were robust to a certain extent. In addition, fewer than 10 articles were included in this study, so Begg’s and Egger’s tests, and meta-regression analyses were not used to analyze the source of heterogeneity (Higgins et al., 2016).

## 4 Discussion

Cerebral small vessel disease is the main cause of cognitive impairment (Pantoni, 2010) and vascular dementia (Zanon Zotin et al., 2021)in the elderly and is an important risk factor for stroke (Elahi et al., 2023). Studies have indicated (Nannoni et al., 2020) that patients with cerebral microbleeds, one of the cerebral small vessel diseases, have significantly impaired executive ability, and processing speed, cerebral microbleeds have a decisive role in vascular cognitive impairment. In a Polish study on cognitive impairment in the middle-aged population, it was stated that patients with severe CSVD were twice as likely to suffer from mild cognitive impairment as those without CSVD (Szcześniak et al., 2020). There is a close correlation between cerebral small vessel disease and cognitive function, which can lead to cognitive impairment. Nowadays, there is an increasing number of studies on biomarkers of CSVD-CI, and the search for biomarkers with high specificity and sensitivity is of great importance for clinical work.

There is a specific type of cerebral small vessel disease that was not included in this study, which is called CADASIL. Cerebral autosomal dominant arteriopathy with subcortical infarcts and leukoencephalopathy (CADASIL) is the most common hereditary CSVD, characterized as a rare genetic disorder in the NOTCH3 gene with non-atherosclerotic and non-amyloid diffuse angiopathy, which mainly leads to brain parenchyma lesions (Yuan et al., 2024). The pathological findings on CADASIL include thinning of blood vessel walls, luminal stenosis, and degeneration of vascular and pericytes (Mizuta et al., 2024). Several promising blood biomarkers such as NOTCH3 extracellular domain (N3ECD), Jagged-1, circulating progenitor cell, Glial fibrillary acidic protein (GFAP), and neurofilament light chain (NFL) have been shown to correlate with CADASIL (Chen C.-H. et al., 2020; Kim et al., 2022). To our knowledge, CADASIL tends to cause migraine, transient ischemic attacks, brain infarcts, cognitive impairment, gait impairment, depression, and so on (Cramer et al., 2024; Finsterwalder et al., 2019). However, we could not catch the potential blood biomarkers for cognitive decline caused by CADASIL in existing studies.

Under inflammation with multiple inflammatory factors, the blood–brain barrier permeability increases and damages the brain endothelial cells, inducing the expression of adhesion molecules and chemokines, which damages the brain tissues (Li et al., 2020). Multiple inflammatory factors of the inflammatory response, such as neutrophil count, neutrophil/lymphocyte ratio (Jiang et al., 2022), hs-CRP, cytokines (Wan et al., 2022), etc. can be used as biomarkers for predicting and evaluating CSVD. Similarly, studies (Wan et al., 2022) have elucidated the close relationship between inflammatory markers including amyloid a, fibrinogen, and cytokines in CSVD. Among the four blood markers analyzed in this study, hsCRP and Lp-PLA2 are related to inflammation and immune function. Hs-CRP is a common non-specific inflammatory indicator with a wide range of applications. hs-CRP is indispensable for predicting respiratory, cardiovascular, rheumatoid, and infection-related diseases as a routine test for hospital admissions. Not only that, hs-CRP is also recognized as an independent risk factor for cognitive impairment after acute ischemic stroke (Guo et al., 2024). A previous cohort study revealed the relationship between hsCRP and cognitive impairment in older adults noted that (Ginsberg et al., 2020): hsCRP levels in older adults over 45 years of age of different populations is a marker of cognitive impairment, but is not yet used to predict the risk of cognitive decline. In this study, we analyzed the difference in serum hsCRP levels between the CSVD-CI group and CSVD without CI group, therefore included 2 papers that showed statistically significant differences between the two groups, with significantly higher hsCRP levels in the CSVD-CI group, which was considered as a possible blood marker of CSVD -CI. Lipoprotein-associated phospholipase A2 (Lp-PLA2) is a secreted enzyme with a molecular weight of 45 k Da that catalyzes acetyl hydrolysis at the sn-2 position of platelet-activating factor and is therefore also known as platelet-activating factor acetylhydrolase. Lp-PLA2 is secreted by macrophages, and mastocytes, binding to plasma low-density lipoproteins (LDL), and high-density lipoproteins (HDL) (Wang L.-M. et al., 2023). Lp-PLA2 can be used as a biomarker for cardiovascular disease, which can be elevated to two times the normal level when stroke and coronary artery disease occur (Khan and Ilies, 2023), and it can also be used as a potentially specific indicator for the diagnosis of early diabetic nephropathy and for observing the progression of diabetic nephropathy (Zhai et al., 2023). It has been demonstrated that Lp-PLA2 can be used as a risk stratifier for chronic heart failure with reduced left ventricular ejection fraction (Jun-Feng et al., 2023), and also a key factor in acute ischemic stroke as an independent risk factor for poor prognosis (Chang et al., 2024). Elevated levels of Lp-PLA2 have been associated with the risk of Parkinson’s disease (Wu et al., 2021). Zuliani et al. showed that Lp-PLA2 activity is elevated in patients with vascular cognitive impairment (Zuliani et al., 2023). In addition, Lp-PLA2 also plays an important role in the prediction of the development of diseases such as atherosclerosis, Alzheimer’s disease, diabetic retinopathy, tumors, etc., especially a promising marker for intracranial atherosclerosis (Huang et al., 2019; Wang Y. et al., 2022). Overall, Lp-PLA2 may be a potential biomarker for neurological disorders, cognitive dysfunction in the available studies. However, the results of our meta-analysis do not yet support Lp-PLA2 as a circulating marker for CSVD-CI, and further exploration is still needed.

Homocysteine (Hcy) is a hydroxyl-containing nonprotein amino acid derived from methionine, a homolog of cysteine. Hcy is associated with a high risk of cerebrovascular disease by mechanisms that oxidative stress, DNA damage, protein sulfation or protein homocysteinylation, triggering of apoptosis, and excitotoxicity (Hermann and Sitdikova, 2021). Hyperhomocysteinemia has been reported to be a risk factor for neurological diseases such as Parkinson’s disease, Alzheimer’s disease, dementia, vascular dementia, and stroke, and is involved in disease progression (Chen S. et al., 2020; Cordaro et al., 2021). Homocysteine is a risk factor for CSVD, and patients with high levels of homocysteine develop a larger volume of WMH (Li et al., 2023). Gao et al. showed that homocysteine is significantly higher in cognitive impairment than those cognitive normal patients and was associated with an increased volume of white matter hyperintensities (Gao et al., 2023). Chen et al. found that Hcy is a risk factor for cerebral microbleeds and lacunes, it also positively correlated with its severity (Chen and Ye, 2022). Similarly, the present meta-analysis showed Hcy as a biomarker of CSVD-CI.

Neurofilament light chain (NfL) is expressed in neuronal axons and stabilizes myelinated axons and provides structural support. When neuronal axons are injured, NfL is markedly elevated in the blood and cerebrospinal fluid (Zanardini et al., 2022). It has been noted that white matter high-density volume is positively correlated with NfL levels. In addition, NfL is also affected by various factors, with aging, males, and Caucasians having higher NfL levels in cerebrospinal fluid (Meeker et al., 2022). Lee et al. categorized into three groups based on baseline serum NfL levels as low, median, and high, demonstrating that high levels of NfL are associated with cognitive stage shifts, and maybe a predictor of cognitive stage shifts (from cognitive normal to mild cognitive impaired, from mild cognitive impaired to dementia)(Lee et al., 2022). NfL can also serve as a biomarker for patients with altered consciousness to differentiate between structural brain diseases such as ischemic stroke, viral encephalitis, and nonstructural brain diseases such as epilepsy and psychiatric disorders (Ongphichetmetha et al., 2023). Previous meta-analysis studies have shown that NfL levels are elevated in the serum of all three types of dementia: Alzheimer’s disease (AD), frontotemporal dementia (FTD), and Creutzfeldt-Jakob disease (CJD) (Gu et al., 2023). However, our meta-analysis results do not yet prove NfL as a biomarker of CSVD-CI, and further studies are still needed to explore the relationship between NfL and CSVD-CI.

The four proteins mentioned above may potentially used as markers of cognitive decline associated with cerebral small vessel disease, but the relationship between these four proteins has not been clearly investigated. Studies show that positive correlations were observed between Hcy and NfL among older adults and there is a clear relationship between inflammatory and neurodegenerative biomarkers (Giudici et al., 2023). Some studies showed the relationship between hs-CRP and homocysteine. Ma, etc. study indicated the postoperative levels of CRP are associated with the incidence and severity of delirium. However, the preoperative levels of homocysteine could modify the association between the postoperative levels of CRP and the incidence of delirium (Ma et al., 2022).

Our findings included different cognitive assessment tools to evaluate cognitive function, which may also increase heterogeneity. A total of 25 of the 29 included studies used MOCA or MMSE to evaluate cognitive functioning. Previous studies have shown that MOCA is a better measure of subtle changes in cognitive capacity due to the lack of ceiling effect with better detection of cognitive heterogeneity than MMSE. However, MMSE is still a most recognized brief cognitive tool (Jia et al., 2021). So both MOCA and MMSE are recommended as widely generalizable tools in various cognitive performances (Chun et al., 2021). Clinical dementia rating (CDR) is a novel assessment tool that can potentially evaluate dementia and facilitate the diagnosis of Alzheimer’s disease (Nosheny et al., 2023). Trail Making Test (TMT) may be possible to screen cognitive physical function for rehabilitation patients, TMT results have a significant correlation with MMSE score (Kubo et al., 2024).

We must mention that this study did not restrict the study population of CSVD to gender or age because they are unchangeable. Still, some studies have pointed out that aging makes the elderly a highly vulnerable population and aging is a major risk factor for CSVD (Li et al., 2020). The prevalence of CSVD increases with age, affecting about 5% of people over 50 years old and almost everyone over 90 years old (Dupré et al., 2024). Although our study does not consider age for CSVD, many studies found that CSVD is a highly age-related disease increasing the risk of stroke and dementia and aging has a significant effect on cerebrovascular diseases including CSVD (Chung et al., 2023). In the available studies, we have not found a substantial difference in CSVD by gender.

## 5 Limitations

This study has limitations: (1) Studies have investigated potential circulating markers for CSVD-related cognitive impairment, but studies combining the biomarkers for both diseases have been limited. The experimental group of the included articles had to be CSVD-CI patients, which might limit the sample size we included. The quality of the included articles varied, meanwhile, the total number of the included articles was relatively small, which might impact the analyzed results of the meta-analysis, restrict the ability to explore the potential confounding factors, or evaluate the heterogeneity across the study. Therefore, we must acknowledge that we provided insufficient quantitative synthesis, to some extent, this study has insufficient generalizability and statistical power. (2) Several factors varied among the studies included in the meta-analysis ranging from biomarker measurement techniques to variations in patient populations. These factors contribute to the complexity of the relationship between biomarkers and study findings. Most of the included articles were cohort and case–control studies, mainly from China, the Netherlands, etc., with a relative lack of regional representation that may impact the generalization of the findings. Markers may change across ethnic, age, and gender populations. (3) As mentioned above, we did not incorporate rare diseases, such as CADASIL into the meta-analysis, which may have caused bias in the data results. (4) Considerable variability in studies of NfL and Lp-PLA2 in studies and lacks adequate validation in independent or diverse populations. The clinical application is uncertain, it is necessary to validate these biomarkers through multi-center trials, cross-sectional studies, longitudinal studies, multi-population studies, prospective studies, and follow-up with patients with CSVD-CI have benefits to achieve the goal of finding accurate blood markers. Further studies are needed to study one or several markers in combination to diagnose CSVD-CI, or, even in combination with cerebrospinal fluid markers. (5) Different cognitive assessment tools may increase the heterogeneity of the study although we developed a careful plan for data extraction. We used SMD to correct the differences in scales between tests, but SMD could not adjust the variation in the effectiveness and accuracy of diverse cognitive assessment tools. Therefore, more standardized tools for cognitive function should be established in the future. (6) We only offer a new perspective on the CSVD-CI study. The study did not adequately address the biomarkers for CSVD-CI because many diseases lead to elevated hsCRP and NfL. All these biomarkers are not specific to CSVD-CI, they can change in states such as tumors, inflammation, injury, stress, the onset of cardiovascular disease, etc. The diagnostic specificity of these markers or other emerging markers of CSVD-related cognitive decline needs to be further explored. (7) Statistical limitations: Some articles only used correlation analyses for biomarkers or did not provide specific numbers that could not be statistically analyzed. As a result, fewer articles were included, which did not meet the basic literature requirements for publication bias in the other three biomarkers studies (≥10 articles), but the presence of publication bias in Hcy analysis.

## 6 Conclusion

In summary, this study provides a promising trend that the hsCRP and Hcy may be used as biomarkers for predicting and diagnosing cognitive impairment in cerebral small vessel disease. While NfL could not draw meaningful conclusions due to the different units of statistical analysis in this meta-analysis, it is not yet possible to conclude that Lp-PLA2 is a potential biomarker of cognitive impairment in cerebral small vessel disease. Due to several limitations encountered during this study such as heterogeneity of cognitive assessment tools, geographic and methodological biases, and limited data for analysis, we could not identify biomarkers with accuracy and specificity for diagnosing CSVD-CI. Therefore, further larger and more diverse cohorts with stronger validity urgently need to validate our preliminary results. Regarding the relationship between NfL, Lp-PLA2, and CSVD-CI, a more reasonable design and a larger number of studies are still needed to verify in the future.
