# Supplementary material for: Potential biomarkers for cerebral small vessel disease with cognitive impairment: a systematic review and meta-analysis
Source: Front Aging Neurosci. 2025 Jan 7;16:1475571. doi: 10.3389/fnagi.2024.1475571 (PMC11747022; doi:10.3389/fnagi.2024.1475571)
Supplement: Supplementary file 1 [file Table_1.docx]

Supplementary Material

# Supplementary material for Literature Quality Assessment

| **Cohort Study** | **Selection** | | | | **Comparability** | | **Assessment** | | | **Total** |
| --- | --- | --- | --- | --- | --- | --- | --- | --- | --- | --- |
|  | Representativeness | Selection of non-exposed | Ascertainment of exposure | Outcome not present at start | Comparability on most important factors | Comparability on other risk factors | Assessment of outcome | Long enough follow-up  (median≥1 year) | Adequacy(completeness) of follow-up |  |
| Harshfield Eric L et al.,2022 (Harshfield et al. 2022) | 1 | 1 | 1 | 1 | 0 | 0 | 1 | 1 | 0 | 6 |
| Hilal Saima et al.,2017 (Hilal et al. 2017) | 1 | 1 | 1 | 1 | 1 | 0 | 1 | 1 | 0 | 7 |
| Ronghui Huang et al.,2023 (Huang et al. 2023) | 1 | 1 | 1 | 1 | 1 | 0 | 1 | 0 | 0 | 6 |
| Salai Kaung H. T.et al.,2023 (Salai et al. 2023) | 1 | 1 | 1 | 1 | 1 | 0 | 1 | 1 | 0 | 7 |
| Xi Tao.et al.,2021(Tao et al. 2022) | 1 | 1 | 1 | 1 | 1 | 0 | 1 | 0 | 0 | 6 |
| Gennip April C. E. van.et al.,2023 (van Gennip et al. 2023) | 1 | 1 | 1 | 1 | 1 | 1 | 1 | 1 | 1 | 9 |

Supplement 1A: Quality of evidence of Cohort studies as reported by Newcastle Ottawa Scale(NOS).

| **Case-control Study** | **Selection** | | | | **Comparability** | | **Assessment** | | | **Total** |
| --- | --- | --- | --- | --- | --- | --- | --- | --- | --- | --- |
|  | suitable | Representativeness of case | Selection of control group | Ascertainment of control group | Comparability on most important factors | Comparability on other risk factors | Ascertainment of  exposure | Same way | Nonresponse |  |
| Laifang Bian et al.,2023 (Bian et al. 2023) | 1 | 1 | 0 | 1 | 1 | 0 | 1 | 1 | 1 | 7 |
| Li Cao et al.,2022 (Cao and Sun 2022) | 1 | 0 | 0 | 1 | 1 | 1 | 1 | 1 | 1 | 7 |
| Yuqin Cao et al.,2019 (Cao et al. 2019) | 1 | 1 | 0 | 1 | 1 | 0 | 1 | 1 | 1 | 7 |
| Xiaohan Chen et al.,2022 (Chen et al. 2023) | 1 | 1 | 0 | 0 | 1 | 0 | 1 | 1 | 1 | 6 |
| Ying Cui et al.,2019 (Cui et al. 2019) | 1 | 1 | 0 | 1 | 1 | 1 | 1 | 1 | 1 | 8 |
| Jingwen Kang et al.,2021 (Kang et al. 2021) | 1 | 1 | 1 | 1 | 1 | 0 | 1 | 1 | 1 | 8 |
| Shasha Liu et al.,2022 (Liu and Fan 2023) | 1 | 1 | 0 | 1 | 1 | 0 | 1 | 1 | 1 | 7 |
| Puttachandra Prabhakar et al.,2017 (Prabhakar et al. 2017) | 1 | 1 | 1 | 1 | 1 | 0 | 1 | 1 | 1 | 8 |
| Wensheng Qu et al.,2022 (Qu et al. 2023) | 1 | 1 | 1 | 1 | 1 | 0 | 1 | 1 | 1 | 8 |
| Fei Wang et al.,2017 (Wang et al. 2017) | 1 | 1 | 0 | 1 | 1 | 0 | 1 | 1 | 1 | 7 |
| Jin Wang et al.,2022 (Wang et al. 2022) | 1 | 1 | 0 | 1 | 1 | 0 | 1 | 1 | 1 | 7 |
| Minghua Wang et al.,2023 (Wang et al. 2023) | 1 | 1 | 0 | 1 | 1 | 0 | 1 | 1 | 1 | 7 |
| Yoo Jun Sang et al.,2020 (Yoo et al. 2020) | 1 | 1 | 0 | 1 | 1 | 0 | 1 | 1 | 1 | 7 |
| Wei Zhang et al.,2023 (Zhang et al. 2023) | 1 | 0 | 0 | 1 | 1 | 0 | 1 | 1 | 1 | 6 |
| Jianhua Zhao et al.,2017 (Zhao et al. 2022) | 1 | 1 | 0 | 1 | 1 | 0 | 1 | 1 | 1 | 7 |
| Weina Zhao et al.,2021 (Zhao et al. 2021) | 1 | 1 | 0 | 1 | 1 | 1 | 1 | 1 | 1 | 8 |
| Shuzhen Zhu et al.,2019 (Zhu et al. 2019) | 1 | 1 | 0 | 1 | 1 | 0 | 1 | 1 | 1 | 7 |

Supplement 1B :Quality of evidence of case-control studies as reported by Newcastle Ottawa Scale(NOS).

| **Cross-sectional study** | **Define the source** | **List inclusion/exclu-sion criteria** | **Indicate time period used for identifying patients** | **Whether subjects were consecutive** | **Whether subjective components were masked** | **Assessments for quality assurance** | **Explain exclusions from analysis** | **Assess or control confound** | **Explain how missing data were handled** | **Summarize response rates and completeness of data** | **Clarify follow-up data** | **Total (0-11)** |
| --- | --- | --- | --- | --- | --- | --- | --- | --- | --- | --- | --- | --- |
| Lu Liu et al.,2022 (Liu et al. 2023) | 1 | 1 | 1 | 1 | not sure | 1 | 1 | 0 | 1 | 1 | 0 | 8 |
| Qianwen Qiu et al.,2022 (Qiu et al. 2022) | 1 | 1 | 1 | 1 | 0 | 1 | 1 | 1 | 1 | 1 | 0 | 9 |
| Xi Tao et al,2021 (Tao et al. 2022) | 1 | 1 | 1 | 1 | not sure | 1 | 1 | 0 | 1 | 1 | 0 | 8 |
| Wada Manabu et al,2010 (Wada et al. 2011) | 1 | 0 | 1 | 1 | not sure | 1 | 1 | 0 | 1 | 1 | 0 | 7 |
| Yanhong Wu et al.,2018 (Wu et al. 2018) | 1 | 1 | 1 | 1 | not sure | 1 | 1 | 1 | 1 | 1 | 0 | 9 |
| Yining Xiao et al,2022 (Xiao et al. 2023) | 1 | 1 | 1 | 1 | not sure | 1 | 1 | 0 | 1 | 0 | 0 | 7 |
| Yang Yanfang et al.,2019 (Yang et al. 2019) | 1 | 1 | 1 | 1 | not sure | 1 | 1 | 1 | 1 | 1 | 0 | 9 |

Supplement 1C: Quality of evidence of cross-sectional studies as reported by The Agency for Healthcare Research and Quality (AHRQ).

# Supplementary Figure





Supplement Figure : Funnel plot of Hcy study showed there some publication bias in included study.
